# Everybody's Page

**Published:** 1889-04-06

**Authors:** 


### Everybody's Page.

Medical Student, to dispensary patient, "I think you must have — a — some kind of a fever; but our class has only got as far as convulsions. Come again next week."

Johann Froscheis, a manufacturer of Nuremberg, has invented pencils, in blue, black, and brown, for writing on the human skin. They are meant to be used for purposes of anatomical and clinical demonstration.

"Most men," said Diogenes, four hundred years before our era, "are within a finger's breadth of being mad. If anyone were to walk along stretching out his forefinger he would seem to be mad; but if he puts out his forefinger he will not be thought so."

A cheap and efficient substitute for the hand-grenades sold for putting out fire can be made by filling ordinary quart bottles with a saturated solution of common salt. The salt forms a coating on everything the water touches, and makes it nearly incombustible.

Lemonade.—Proper home-made lemonade, got from real lemons, is one of the most wholesale, as well as pleasant, drinks known; in fact, lemon juice in any form is of great value. As is well known, it is an admirable anti-scorbutic; it may also be used in intermittent fevers, mixed with strong black coffee, and has had beneficial results in malarial fevers. Used externally it keeps the hands white and soft, helps to prevent chilblains, and is said to cure warts.

St. Hubert, the patron saint of hunters, seems to have been the Pasteur of his day—to have bequeathed his power to his clothes. The lining of his jerkin, which is preserved in the chapel at Royaumont, near Chantilly, is said to have the power of curing hydrophobia. The lining is made of coarse canvas, not unlike sail-cloth, and when a sufferer who has been bitten by a dog comes for cure, a portion of a thread is drawn from the lining and laid on the wound caused by the dog's teeth. A bandage is then applied, and when a certain number of prayers and litanies have been said the cure is complete.

Chemists often have strange requests sent to them by people who insist on diagnosing their own ailments, and trying to prescribe for them. Sometimes it is easy to find out what the customer means, as for example, the request for "some of the essence you put people to sleep with when you cut their fingers off," though that seems a long way round for ether or chloroform, and "an ounce of the smelling stuff that goes through your brain," cannot well mean anything but ammonia. But when it comes to "something for a sore baby's eye," or "a plaster for a man kilt with stitches," or "something for a woman with a bad cough and cannot cough," or, finally, "something, I forget the name, but it is for a cure," what, in the name of the pharmacopœia, is a poor dispensing chemist to do?

China is one of the most self-supporting of countries. Three-fifths of the population live almost entirely on rice, and the remainder on millet and wheat, and the greater part of the soil is devoted to the cultivation of these grains. Maize is being more and more cultivated, and a special glutinous kind of rice, called Hwangmi, from which an undistilled wine is made, is a highly valued crop. About a thousand years ago, the Chinese condescended to import spinach from Persia, and the name by which it is called, "pot say," signifies the Persian vegetable. Lately they adopted the European beetroot, and though cannot bring their minds to accept railways and electric telegraphs, they are not above being converted to the use of many European vegetables.

"Look here, doctor," said the patient, who was just recovering from typhoid fever, and didn't like the limits set to his diet; "look here, I wo'n't stand it any longer. It isn't worth while to starve to death for the sake of living a little longer."

The paradise of railway travelling must be Lower Hungary, where the companies are planting hedges of Provence roses to protect the cuttings from drifting snow. Think of journeying in a land where the smoke and dust of the engine are atoned for by the proximity of miles of blooming fragrant roses.

Sponges are found chiefly in the Mediterranean and Red Seas, around the Carribean Islands, and the Bahamas, and off the west and south-west coasts of Florida. A new kind has recently been found in Australian waters, and another on the Atlantic coast of Brazil, but these are of a coarser kind, and of less commercial value.

A simple method of cleaning and polishing floors is to rub them every morning with a large flannel cloth, which is soaked in paraffin every fortnight or so. After wiping well with the cloth, brush briskly up and down the planks with a stiff broom, and after a few days of this treatment the floor will take and retain an admirable polish.

Very often it is not the necessary preaching that causes what is called "clergyman's sore throat," but a sedentary life, errors in diet, and ignorance of the proper method of using the voice. We do not hear of actor's, singer's, or lecturer's sore throat, although these make as much use of the vocal organs as the average clergyman, if not more.

Occasionally one sees a paragraph in the newspapers headed "What Mr. So-and-So drinks," or occasionally the supposed pet beverage of a whole profession is advertised, as if, because a successful dramatist likes cold tea, the consumption of cold tea was a recipe for growing Shakespeares. Acting on this principle, we mean to give a little information as to "What Parisians drink." They drink Seine water, and out of the Seine there were fished, last year, the following number of dead animals: 2,021 dogs, 977 cats, 2,257 rats, 507 chickens and ducks, 3,066 kilos of butcher's refuse, 210 rabbits and hares, 10 sheep, 2 horses, 71 pigs, 49 geese and turkeys, 10 calves and goats, 3 monkeys, 1 snake, 2 squirrels, 3 porcupines, 1 parrot, 609 birds of various kinds, 3 foxes, 130 pigeons and partridges, 3 hedgehogs, 8 peacocks, and 1 seal. A perusal of this list will probably deter visitors to the coming Paris Exhibition from drinking much water during their stay.

"How to get read" is one of the problems of the day—even so much more pressing to many of us than how to read. Aspiring authors want to be read, aspiring advertisers want to be read, aspiring promoters of all sorts of companies and associations want also to be read, and this is how they manage it. In the days of their youth and comparative innocence they simply send out their reports and circulars in the ordinary stamped wrapper. If this brings in no result, and they have reason to surmise that the interesting document has been consigned to the wastepaper basket, they send out another copy with "private" scribbled on the wrapper; the next superscription is "immediate," then comes "pressing," then "important," and so on through a long and varied range of adjectives, and in the end some simple-minded person does come to believe that the "private" report of the "Association for Aiding the Amelioration of Central Australasia" demands immediate attentions, and deals with a question of "pressing importance."

